# Antioxidant Activity of *Pistacia vera* Fruits, Leaves and Gum Extracts

**Published:** 2012

**Authors:** Hossein Hosseinzadeh, Sayyed Abolghasem Sajadi Tabassi, Negar Milani Moghadam, Marzieh Rashedinia, Soghra Mehri

**Affiliations:** a *Pharmaceutical Research Center, Pharmacodynamy and Toxicology Department, School of Pharmacy, Mashhad University of Medical Sciences, Mashhad, Iran.*; b *Department of Pharmaceutics, Pharmaceutical Research Center for Medicinal Plants, School of Pharmacy, Mashhad University of Medical Sciences, Mashhad, Iran.*; c *Pharmacodynamy and Toxicology Department, School of Pharmacy, Mashhad University of Medical Sciences, Mashhad, Iran.*

**Keywords:** *Pistacia vera*, Pistachio, Antioxidant, Deoxyribose, Free radical, Lipid peroxidation

## Abstract

The side effects of synthetic antioxidants have been considered in different studies. Accordingly, there is an increasing interest toward the use of natural substances instead of the synthetic ones. In this study, the aqueous and ethanolic extracts of *Pistacia vera *leaves and fruits as well as hydroalcoholic extract of gum were tested for a possible antioxidant activity using in vitro methods. Deoxyribose assay, erythrocyte membrane lipid peroxidation and liver misrosomal non- enzymatic lipid peroxidation tests were used as an *in-vitro *model for determination antioxidant activity. The extract were evaluated at different concentratios: 25,100, 250, 500 and 1000 μg/mL. In all procedures, all extracts showed free radical scavenging activity. The effect of ethanolic extract of *P. vera *fruit at 1000 μg/mL was quite similar to positive control (DMSO 20 mM) in deoxyribose method. In two other tests, the ethanolic extracts of fruits and leaves were more effective than the aqueous extracts to inhibit malondialdehyde generation. Phytochemical tests showed the presence of flavonoids and tannins in *Pistocia vera *extracts. The present study showed that extracts of different part of *P. vera *have antioxidant activity in different in vitro methods. The ethanolic extracts of leaves and fruits showed more roles for antioxidant properties and gum hydroalcoholic extract demonstrated less antioxidant effect.

## Introduction

It has been well established that oxidative stress plays an important role in the onset of different diseases, including atherosclerosis, rheumatoid arthritis, cancer and the degenerative diseases associated with aging (1, 2). Consequently, the supplement of dietary antioxidants will help to attenuate the damage of the body induced by oxidative stress, and can be used as potential therapeutic or preventive drugs for the risk of many free radical-mediated diseases.

In the last few decades, the natural antioxidant that may be obtained from different plant parts, flavonoids and poly phenolic compounds are paid more attention to, because phenolic compounds isolated from plants can act as free radical scavengers, metal chelators, and singlet oxygen quenchers (3).

*Pistacia vera *L., is a plant member of Anacardiaceae family and native to Asia. Pistachio nut is mostly produced in Iran and some other countries (4). *Pistacia *species have caught up the interest of researchers due to the study on different part of this plant such as leaves, kernels, hulls and gum demonstrate various biological activities such as antioxidant potential, antimicrobial, anti-inflammatory, mainly due to flavonoids and other phenolic components and anti-insect activities (5-11). It has been proved that Pistachio nuts are a rich source of phenolic compounds and have been considered because of high antioxidant potential (12).

Investigation on pistachio green hull has showed antioxidant, anti-microbial and antimutagenic activity (13). A clinical trial study on young men demonstrated that a pistachio diet improved blood glucose level, endothelial function, and some indices of inflammation and oxidative status (14). Also *P. vera *L. gum extract demonstrated a protective effect on oxidative damage in rat cerebral ischemia-reperfusion (15). It is also showed that a gum extract has antinocieptive and anti-inflammatory effect (16). In other study *P. vera *L. (Pistachio) leaves and nuts aqueous extracts showed antiemetic effect in young chicken (17). 

In recent studies, the side effects of synthetic antioxidants are considered. There is an increasing interest to the use of natural substances instead of the synthetic ones. The purpose of this study was the evaluation of antioxidant effects from different parts of pistachio fruits, namely leaves and gum using three *in-vitro *approaches: deoxyribose assay, erythrocyte membrane peroxidation and rat liver microsomal lipid peroxidation induced by Fe^2+^/ascorbate.

## Experimental

Ascorbic acid, Deoxyribose, Tiobarbitoric acid (TBA), ferric chloride, methanol, butylated hydroxytoluene (BHT) and trichloroacetic acid (TCA) were obtained from Merck (Germany).

*Plant material and Preparation of extracts*

*P. vera *L. was collected from (Khorasan- Gonabad region) I.R Iran. It was identified by Mr. Ahi in the Herbarium of School of Pharmacy, Mashhad University of Medical Sciences (MUMS), IR. Iran. For the decoction extract, 1 L water was added to 100 g plant material and boiled for 15 min and percolation was performed till the solvent become colorless. Afterwards, the solution was filtrated and evaporated in a water bath (maintained at 30-40°C). The extract was stored in a refrigerator at 4°C.

To obtain the ethanolic extract, the leaves and fruits of plant were prepared by defatted powder via Soxhlet with petroleum ether giving as dry residue and was macerated in ethanol 80° (v/v) for 72 h. Then, the macerated mixture was filtered and evaporated as mentioned previously.

The gum was extracted from the resin by cold maceration by hydrodistillation with ethanol. The combined hydroalcoholic extract was filtered through filter paper and evaporated to dryness on water bath.

*Characterization of extract by HPLC*

The separation was carried out on a millipore column (5 μm, 1.5 X 3.9 mm) using gradient elution. Gradient was performed using water-phosphoric acid (0.1 N, 99:1) and acetonitrile-phosphoric acid (0.1 N, 99:1) at a total flow rate of 1 mL/min; gradient composition (min, % acetonitrile-phosphoric acid): 0.0, 5; 5.0, 7.5; 10, 10; 15, 12.5; 20, 15; 25, 17.5; 30, 20; 35, 22.5; 40, 25). The extracts were dissolved in methanol and filtered through a membrane filter (0.45 μm). 1.0 μL sample of 10 g/L of extract was injected in to a reversed-phase column (RPC-18). The peaks were momtored at 236 nm.

*Animals*

Male Wistar rats weighing 200-250 g were used for the study. The animals were bred and housed in the Animal House of the Faculty of Pharmacy Mashhad University of Medical Sciences accordance with ethical committee Acts.

*Deoxyribose assay*

In this method, after mixing the materials, hydroxyl radicals were produced by ascorbic acid, H_2_O_2_ and Fe^3+^-EDTA thus deoxyribose degradation take placed and produced malondialdhyde. The reaction mixture contained 100 μL of 28 mM 2-deoxy- 2-ribose 500 μL solution of various concentration of the material test (aqueous and ethanolic extracts of pistachio fruits and leaves and hydroalchoholic extracts of gum in buffer), 200 μL of 200 μM FeCl_3_ and 1.04 mM EDTA (1:1 v/v), 100 μL H_2_O_2_ (1 mM) and 100 μL ascorbic acid (1 mM). All solutions were prepared freshly. After an incubation period of 1 h at 37°C, 1 mL of TBA (1%in 50 mM NaOH) and 1 mL of TCA were added to the reaction Mixture and the tubes were heated at 100°C for 20 min. The degree of deoxyribose degradation was measured by the TBA reaction. Absorbance was read at 532 nm (18, 19). The percentage Absorbance was read at 532 nm. The percentage of inhibition of deoxyribose degradation was calculated using the following equation:

%Inhibition = (A_0_ - A_1_) / A_0_ × 100

Here, A_0_ is the absorbance of the control in the absence of samples; A_1_ is the absorbance in the presence of samples. DMSO was used as a positive control (20) and the negative control was all of the reaction mixture without extracts.

*Site-specific reactions assay*

This assay was prepared in the following three ways:

This test was done as mentioned previous to except that 100 mL of FeCl_3_ instead of 200 mL of Fe^3+^-EDTA Solution was extra, for evaluated potency of sample radical scavenging and Fe chelating.

The evaluation was performed without ascorbic acid as a starter for oxidation.

Deoxyribose itself was omitted from the test (18).

*Erythrocyte membrane peroxidation*

According to the method that was described before (19, 21), the evaluation of protective effects of pistachio extracts, were done with modification. After anesthizing the Wistar rats (200-250 g) with chloroform, whole blood were collected via a cardiac puncture to heparinized tubes. The RBC was separated from plasma by centrifugation at 1500 g for 15 min. Packed RBC was washed twice with NaCl 0.15 M, and preincubated with phosphate buffer (pH = 7.4) containing sodium azide (1 mM) to inhibit catalase. Then H_2_O_2_ (10 mM) was added, peroxidation was initiated H_2_O_2 _(10 mM). 100 μL solutions of various concentrations of the material test were added and the mixture was incubated at 37°C for 60 min. The addition of 28% (w/v) trichloroacetic acid terminated reaction. Lipid peroxidation was measured using thiobarbitoric method. The quantity of MDA was determined by measuring the absorbance at 532 nm BHT was used as a positive control (21, 22).

*Liver microsomal preparation and lipid peroxidation induced by Fe* ^2+^ */ascorbat*

This test was carried out according to the method that was described previously (23). Wistar rats were anesthetized and liver was perfused with ice-cold saline through the portal vein until getting uniformly pale and were immediately removed. Then, pieces of liver were homogenized with 4 volume of ice-cold 0.1 M potassium phosphate buffer (pH = 7.4) containing 1.15% (w/v) KCl. The homogenate was centrifuged at 10000 g for 60 min. The supernatant was used for the study. According to the mentioned previous method, (23) for measuring antioxidant activity, rat liver microsome (2 mg/ mL) was mixed with 0.1 mL of FeSO4 (26% mM), 0.1 mL of ascorbate (0.13 mM), 0.1 mL of the sample in 150 mM KCl/Tris-HCl buffer solution (pH = 7.4). The mixture was incubated at 37°C for 60 min in a water bath; 0.75 mL of 2 M trichloroacetic acid/1.7 M HCl was added to stop trhe reaction, then tubes were centrifuged (4000 rpm, 10 min) and 0.5 mL of the supernatant was mixed with 0.15 mL TBA and was heated at 95°C for 10 min. The level of malondialdehyde was determined by measuring the absorbance at 532 nm. The percent of lipid peroxidation inhibition was calculated by following Equation:

%I = (A_0_ - A_1_ / A_0_) × 100 

Here, A_0_ is the absorbance of the control reaction; A_1_ is the absorbance in the presence of the agents. BHT was used as a positive control (20).

*Phytochemical test*

Phytochemical screening of the extract was performed using the following reagents and chemicals (24): Alkaloids with Dragendorff’s reagent, flavonoids by the use of Mg and HCl; tannins with 1% gelatin and 10% NaCl solutions and saponins with ability to produce hemolysis of RBC.

*Statistical analysis*

Statistical analysis was performed using one-way ANOVA followed by Tukey-Kramer post hoc test for multiple comparisons. The p-value less than p < 0.05 were considered to be statistically significant. PCS software was used to calculate IC_50_ value.

## Results and Discussion

*Yield of extraction*

Yield of extraction was 4.22% for leaves and 10% for fruits. In ehanolic extraction method, yield of extraction for leaves and fruits were 26% and 6.9% respectively. For Pistachio gum, the yield was 6.9%.

*Deoxyribose degradation assay*

In deoxyribose degradation method, absorption spectra at 532 nm were evaluated for extracts in various concentration (25, 100, 250, 500 and 1000 μg/mL) and blank was used. All of tested compound showed antioxidant activity and OH radical scavenging effect (p < 0.001 VS control). The IC_50_ values of aqueous and ethanolic extracts of fruits were149.2 μg/mL and 64.7 μg/mL and for leaves 105.4 μg/mL and 84.8 μg/mL, respectively and for gum extract was 285.5 μg/mL. The results are shown in Table 1. In deoxyribose assay ability of radical scavenging and inhibition of deoxyribose degradation (18), all exracts inhibited MDA production in a dose-dependent manner. The effect of fruit ethanolic extract (1000 μg/mL) was similar to positive control.

**Table1:** Free radical scavenging activity of *Pistacia vera *extracts in deoxyribose assay

**Concentr Concentration ** **(μg/mL) ation ** **(μg/mL)**	**%Inhibition**				
	**Ethanolic fruit extract**	**Aqueous fruit extract**	**Ethanolic Leaf extract**	**Aqueous Leaf extract**	**Hydroalcholic Gum extract**
Control	-	-	-	-	-
25 (μg/mL)	38.6***	24.2***	34.3***	27.4***	14.7***
100	53***	37.5***	54.9***	52.7***	34.1***
250	64.6***	63***	56.6***	59.1***	46.2***
500	85.3***	70.7***	72.8***	73***	63.5***
1000	90.3***	77.1***	88.5***	84.5***	67.4***
DMSO(20 mM) positive control	91.5***	91.8***	90.9***	91.3***	91.3***

% Inhibition = A control (532) - Asample (532) / A control (532) × 100 *** p < 0.001, as compared to control

**Table 2:** Results of the assay for site-specific reactions of the ethanol and aqueous extracts of fruit, leaf and gum of of *Pistacia vera*

**Sample **	**Concentration **	**Omit EDTA **	**Omit Vit C **	**Omit deoxyribose**
Ethanolic fruit extract	Negative control	0.617	0.156	0.084
	1000μg/mL	0.099	0.210	0.037
	500 μg/mL	0.163	0.170	0.030
	250 μg/mL	0.256	0.144	0.024
	100 μg/mL	0.340	0.127	0.019
	25 μg/mL	0.426	0.102	0.009
Aqueous fruit extract	Negative control	0.617	0.156	0.082
	1000μg/mL	0.114	0.143	0.064
	500 μg/mL	0.167	0.141	0.056
	250 μg/mL	0.297	0.129	0.027
	100 μg/mL	0.470	0.108	0.009
	25 μg/mL	0.326	0.077	0.002
Ethanolic Leaf extract	Negative control	0.609	0.159	0.079
	1000μg/mL	0.111	0.197	0.069
	500 μg/mL	0.178	0.149	0.061
	250 μg/mL	0.262	0.152	0.032
	100 μg/mL	0.419	0.116	0.027
	25 μg/mL	0.487	0.103	0.015
Aqueous Leaf extract	Negative control	0.609	0.159	0.079
	1000μg/mL	0.171	0.168	0.058
	500 μg/mL	0.210	0.140	0.037
	250 μg/mL	0.293	0.112	0.019
	100 μg/mL	0.327	0.098	0.016
	25 μg/mL	0.402	0.061	0.007
Hydroalcholic Gum extract	Negative control	0.609	0.159	0.083
	1000μg/mL	0.236	0.131	0.056
	500 μg/mL	0.280	0.119	0.045
	250 μg/mL	0.341	0.117	0.027
	100 μg/mL	0.399	0.081	0.021
	25 μg/mL	0.419	0.042	0.010

*RBC lipid peroxidation*

High polyunsaturated fatty acid contents cause membrane lipids particularly susceptible to oxidative damage (21, 25). Lipid peroxidation is one of the important reactions that induced by oxidative stress (26, 27).

TBARS were expressed with Pmol MDA produced in the presence of different concentrations of extracts. The IC_50_ values of the aqueous and ethanolic extracts of fruits were 768.3 μg/mL and 325.1 μg/mL and for leaves were 314.5 μg/mL and 231.4 μg/mL, respectively and gum extract showed antioxidant effect less than 50%. Results are shown in Tables 3-5.

All extracts prevented oxyradical generation. The fruits ethanolic extract was more effective than aqueous extract probably due to existence of linoleaic and linolenic fatty acids. The antioxidant effect of pistachio fruits (28, 29) was due to antioxidant effects of phenolic compounds (flavonoids and tannin) (30). It was shown that Pistachio skins has better antioxidant activity i compared with seeds in different tests such as (DPPH assay, Folin-Ciocalteau colorimetric method and TEAC assay, SOD-mimetic assay). The excellent antioxidant activity of pistachio skins can be due to its higher content of antioxidant phenolic compounds (31). In another study *P. lentiscus *resin showed antioxidant activity, Fe^2+^ chelating activity and it inhibited inflammation (32).

**Table 3:** Effects of ethanolic and aqueous extracts of *Pistacia vera *fruits in RBC lipid peroxidation assay

**Concentration **	**Aqueous extract**			**Ethanolic extract**		
	**MDA**	**p**	**Inhibition%**	**MDA**	**p**	**Inhibition%**
Control	365.38 ± 9.26	-	-	385.79 ± 6.52	-	-
25 (μg/mL)	360.9 ± 4.13	Ns	1.2	366.62 ± 9.26	Ns	5
100	339.35 ± 4.65	Ns	7.1	339.34 ± 5.72	0.001	12
250	255.48 ± 6.27	0.001	30	192.56 ± 7.26	0.001	50
500	211.94 ± 3.64	0.001	42	125.91 ± 9.38	0.001	67.4
1000	178.8 ± 9.56	0.001	51	96.42 ± 3.64	0.001	75
BHT(0.04) mM	74.34 ± 2.15	0.001	79.6	78.18 ± 2.25	0.001	80

Vlues are mean ± SEM (n=6). TBARS were expressed with pmol MDA produced in the presence of different concentrations of extracts. ns: not significant. BHT: Possitive control

**Table 4:** Effects of ethanolic and aqueous extracts of Pistacia vera leaves in RBC lipid peroxidation assay

**Concentration **	**Aqueous extract **			**Ethanolic extract **		
	**MDA**	**p**	**Inhibition%**	**MDA**	**p**	**Inhibition%**
Control	377.15 ± 9.33	-	-	402.06 ± 3.4	-	-
25 (μg/mL)	356.9 ± 6.22	Ns	5.4	351.41 ± 10.51	Ns	12.6
100	298.81 ± 5.38	0.001	20.8	291.01 ± 6.82	0.001	28
250	214.23 ± 9.15	0.001	43.2	233.3 ± 5.24	0.001	42
500	109.22 ± 5	0.001	71	92.601 ± 3.29	0.001	77
1000	106.23 ± 3.65	0.001	71.8	88.93 ± 4.97	0.001	77.9
BHT (0.04) mM	80.7 ± 1.91	0.001	78.6	77.25 ± 2.75	0.001	80.8

Vlues are mean ± SEM (n=6). TBARS were expressed with pmol MDA produced in the presence of different concentrations of extracts. ns: not significant.

BHT: Possitive control.

**Table 5:** Effects of hydroalcoholic extracts of Pistacia vera gum in RBC lipid peroxidation assay

**Concentration **	**MDA**	**p**	**Inhibition%**
Control	371.6 ± 6.49	-	-
25 (μg/mL)	363.51 ± 4.06	Ns	2.1
100	349.86 ± 8.22	Ns	5.8
250	316.31 ± 5.78	0.001	14.9
500	203.49 ± 7.42	0.001	45.2
1000	196.74 ± 7.56	0.001	47
BHT (0.04) mM	75.78 ± 2.66	0.001	79.6

Vlues are mean ± SEM (n=6). TBARS were expressed with pmol MDA produced in the presence of different concentrations of extracts. ns: not significant.

BHT: Possitive control.

*Liver microsomal lipid peroxidation*

Treatment of liver microsomal with extracts was produced a significant decrease of MDA generation as compared with control treatment. The IC_50_ values of aqueous and ethanolic extracts of fruits 1441.5 μg/mL and 648.7 μg/mL, and leaves were 1101.1 μg/mL and 700.1 μg/mL, respectively and gum extract showed antioxidant effect less than 50%. Results are shown in Tables 6-8.

**Table 6:** Effects of ethanolic and aqueous extracts of Pistacia vera fruits in microsomal lipid peroxidation assay

		**Aqueous extract **			**Ethanolic extract **		
**Concentration **	**MDA**		**p**	**Inhibition%**	**MDA**	**p**	**Inhibition%**
Control	1.98 ± 0.083		-	-	1.89 ± 0.073	-	-
25 (μg/mL)	1.84 ± 0.074		Ns	7.2	1.66 ± 0.044	Ns	12.1
100	1.53 ± 0.027		0.001	22.7	1.37 ± 0.049	0.001	27.6
250	1.49 ± 0.043		0.001	24.7	1.29 ± 0.034	0.001	31.4
500	1.34 ± 0.074		0.001	32.6	1.04 ± 0.063	0.001	44.9
1000	1.051 ± 0.076		0.001	47.1	0.77 ± 0.096	0.001	59.5
BHT (0.04) mM	0.55 ± 0.02		0.001	72.2	0.53 ± 0.052	0.001	71.8

Vlues are mean ± SEM (n=6). TBARS were expressed with pmol MDA produced in the presence of different concentrations of extracts. ns: not significant. BHT: Possitive control

**Table 7:** Effects of ethanolic and aqueous extracts of Pistacia vera leaves in microsomal lipid peroxidation assay

**Concentration **	**Aqueous extract **			**Ethanolic extract **		
	**MDA**	**p**	**Inhibition%**	**MDA**	**p**	**Inhibition%**
Control	1.98 ± 0.08	-	-	1.89 ± 0.073	-	-
25 (μg/mL)	1.80 ± 0.06	Ns	9.1	1.79 ± 0.063	Ns	5.4
100	1.67 ± 0.05	0.05	15.9	1.57 ± 0.08	0.05	16.9
250	1.61 ± 0.06	0.01	18.7	1.32 ± 0.056	0.001	30.7
500	1.07 ± 0.07	0.001	45.8	1.02 ± 0.043	0.001	46.1
1000	0.97 ± 0.06	0.001	51	0.84 ± 0.094	0.001	55.2
BHT (0.04) mM	0.55 ± 0.02	0.001	72.2	0.53 ± 0.052	0.001	71.8

Vlues are mean ± SEM (n=6). TBARS were expressed with pmol MDA produced in the presence of different concentrations of extracts. ns: not significant. BHT: Possitive control

**Table 8:** Effects of hydroalcoholic extracts of Pistacia vera gum in microsomal lipid peroxidation assay

**Concentration **	**MDA**	**p**	**Inhibition%**
Control	1.89 ± 0.05	-	-
25 (μg/mL)	1.78 ± 0.04	Ns	6
100	1.74 ± 0.02	Ns	8.1
250	1.66 ± 0.07	0.05	12.6
500	1.37 ± 0.06	0.001	27.4
1000	1.24 ± 0.05	0.001	34.5
BHT (0.04) mM	0.53 ± 0.04	0.001	72

Vlues are mean ± SEM (n=6). TBARS were expressed with pmol MDA produced in the presence of different concentrations of extracts. ns: not significant. BHT: Possitive control

*Phytochemical tests*

**Figure 1:**
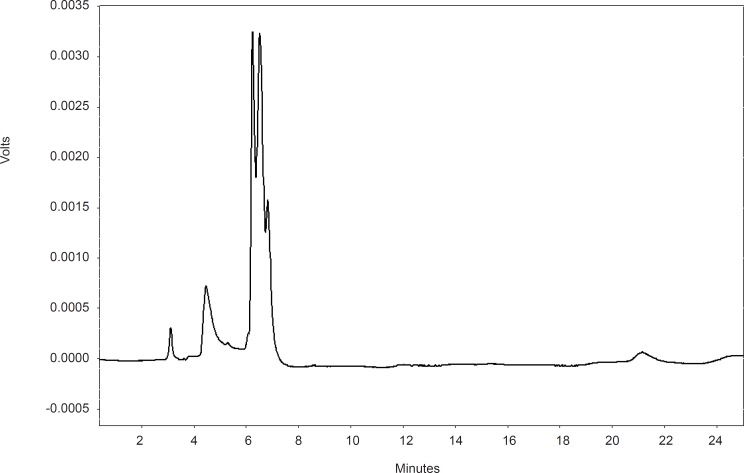
HPLC fingerprint of P. vera ethanolic gum extract

All extracts were negative as for the existence of alkaloid. The aqueous and ethanolic extracts of fruits were negative for tannin content but both extracts of leaves and gum were positive in tannin test. The aqueous and methanolic extracts of fruits had considerable amount of flavonoids but leaves ethanolic extracts and gum extracts had fewer amount. Saponin was not found in the extracts. According to the result of phytochemical tests in this research, the protective effect of extract is probably due to the presence of tannin in gum and leaves extract and flavonoids in fruit extract. HPLC fingerprints of the extract indicated five peaks (Figure 1).

## Conclusion

The present study showed that extracts of different part of *P. vera *have antioxidant activity in different in vitro methods. The ethanolic extracts of leaves and fruits showed more roles for antioxidant properties and gum hydroalcoholic extract demonstrated less antioxidant effect.
